# Role of MSX1 in Osteogenic Differentiation of Human Dental Pulp Stem Cells

**DOI:** 10.1155/2016/8035759

**Published:** 2016-08-28

**Authors:** Noriko Goto, Katsumi Fujimoto, Sakiko Fujii, Hiroko Ida-Yonemochi, Hayato Ohshima, Takeshi Kawamoto, Mitsuhide Noshiro, Chisa Shukunami, Katsuyuki Kozai, Yukio Kato

**Affiliations:** ^1^Department of Pediatric Dentistry, Institute of Biomedical & Health Sciences, Hiroshima University, 1-2-3 Kasumi, Minami-ku, Hiroshima 734-8553, Japan; ^2^Department of Dental and Medical Biochemistry, Institute of Biomedical & Health Sciences, Hiroshima University, 1-2-3 Kasumi, Minami-ku, Hiroshima 734-8553, Japan; ^3^Department of Molecular Biology and Biochemistry, Institute of Biomedical & Health Sciences, Hiroshima University, 1-2-3 Kasumi, Minami-ku, Hiroshima 734-8553, Japan; ^4^Department of Dental Science for Health Promotion, Institute of Biomedical & Health Sciences, Hiroshima University, 1-2-3 Kasumi, Minami-ku, Hiroshima 734-8553, Japan; ^5^Division of Anatomy and Cell Biology of the Hard Tissue, Department of Tissue Regeneration and Reconstruction, Niigata University Graduate School of Medical and Dental Sciences, 2-5274 Gakkocho-dori, Chuo-ku, Niigata 951-8514, Japan

## Abstract

Msh homeobox 1 (MSX1) encodes a transcription factor implicated in embryonic development of limbs and craniofacial tissues including bone and teeth. Although MSX1 regulates osteoblast differentiation in the cranial bone of young animal, little is known about the contribution of MSX1 to the osteogenic potential of human cells. In the present study, we investigate the role of MSX1 in osteogenic differentiation of human dental pulp stem cells isolated from deciduous teeth. When these cells were exposed to osteogenesis-induction medium, runt-related transcription factor-2* (RUNX2)*, bone morphogenetic protein-2* (BMP2)*, alkaline phosphatase* (ALPL)*, and osteocalcin* (OCN)* mRNA levels, as well as alkaline phosphatase activity, increased on days 4–12, and thereafter the matrix was calcified on day 14. However, knockdown of* MSX1* with small interfering RNA abolished the induction of the osteoblast-related gene expression, alkaline phosphatase activity, and calcification. Interestingly, DNA microarray and PCR analyses revealed that* MSX1* knockdown induced the sterol regulatory element-binding protein 2* (SREBP2)* transcriptional factor and its downstream target genes in the cholesterol synthesis pathway. Inhibition of cholesterol synthesis enhances osteoblast differentiation of various mesenchymal cells. Thus, MSX1 may downregulate the cholesterol synthesis-related genes to ensure osteoblast differentiation of human dental pulp stem cells.

## 1. Introduction 

Msh homeobox 1 (MSX1) is a homeobox transcriptional factor involved in limb-pattern formation and craniofacial development and specifically in odontogenesis. Mouse* Msx1* mutations cause craniofacial malformation and tooth agenesis [1].* Msx1*-knockout mice show arrested tooth development at the bud stage and embryonic lethal defects [2]. Msx1 is expressed at high levels in craniofacial skeletal cells during early postnatal development [3], and transgenic mice expressing* Msx1* under the control of the alpha (I) collagen promoter exhibit increased osteoblast number, cell proliferation, and apoptosis [4], suggesting Msx1 may have a role in craniofacial bone modeling. MSX1 is also expressed at high levels in the dental mesenchyme at the cap and bell stages [5] and may be a suppressor for cell differentiation that maintains mesenchymal cells in a proliferative state to ensure robust craniofacial and tooth development [6]. In addition, MSX1 is an upstream and downstream regulator for the bone morphogenetic protein BMP2/BMP4 signaling pathway [7, 8]. Mutations in human* MSX1* also cause cleft lip/palate and tooth agenesis [9, 10]. However, the role of MSX1 in human craniofacial and tooth development has not been fully understood.

Dental pulp stromal cells isolated from whole pulp tissue can differentiate into osteoblasts, odontoblasts, endothelial cells, nerve cells, and adipocytes* in vitro*. Some of these cells identified by several cell surface antigens are referred to as dental pulp stem cells (DPSCs) [11, 12]. DPSCs may play a role in dentinogenesis/osteogenesis in both developing and injured teeth. Furthermore, these cells are a promising source of cell-based regenerative therapies for dental, skeletal, vascular, and neuronal diseases [13, 14]. Human DPSCs (hDPSCs) have not been fully characterized at the molecular level, but a previous reported showed that* MSX1* is expressed at higher levels in hDPSCs than in bone marrow-derived mesenchymal stem cells and fibroblasts [15]. MSX1 may participate in the control of primary or secondary dentin formation and reparative dentin or osteodentin/bone formation in injured pulp tissue, in addition to the physiological role such as the maintenance of dental pulp stem/progenitor cells in healthy teeth. In the present study, we explored the role of MSX1 in pulpal mesenchymal cells using human DPSCs in culture.

Statins are a class of drugs that function as specific inhibitors of 3-hydoroxy-3-methylglutaryl-CoA (HMG-CoA) reductase, a rate-limiting enzyme in cholesterol synthesis. Numerous studies have shown that statins exert bone anabolic effects in osteoblasts and osteogenic precursor cells [16, 17]. Simvastatin enhances alveolar bone remodeling in the tooth extraction socket [18], enhances bone fracture healing [19], and reduces alveolar bone loss and tooth mobility in chronic periodontitis [20]. In addition, simvastatin enhances odontoblast/osteoblast differentiation of DPSCs and mesenchymal stem cells isolated from other tissues [17, 21, 22]. These studies indicate a close relationship between cholesterol synthesis and osteoblast differentiation.

Here, we demonstrated the role of MSX1 in osteoblast differentiation and cholesterol synthesis in hDPSCs using small interfering RNA (siRNA) against* MSX1*. DNA microarray analyses revealed that knockdown of* MSX1* in hDPSCs undergoing osteogenic differentiation abolished the expression of various osteoblast-related genes but enhanced the expression of cholesterol synthesis-related genes. Our results suggest that MSX1 enhances osteoblast differentiation and calcification in hDPSCs through repression of cholesterol synthesis genes and induction of osteoblast-related genes.

## 2. Material and Methods 

### 2.1. Human DPSCs

Extracted healthy deciduous teeth were collected from 6–12-year-old children following protocols approved by the ethical authorities at Hiroshima University (permit number: D88-2). Written informed consent was obtained from the subject or subject's parent. Pulp tissue specimens from deciduous teeth were minced and digested with 3 mg/mL collagenase type I (Life Technologies, Carlsbad, CA, USA) and 4 mg/mL dispase (Roche Diagnostics, Mannheim, Germany) in Dulbecco's modified Eagle's medium (DMEM; Sigma, St. Louis, MO, USA) for 1 h at 37°C. Single cell suspension was obtained by passing cells through a 70 *μ*m cell strainer (CORNING, Corning, NY, USA). The cells were incubated in DMEM supplemented with 20% fetal bovine serum (FBS; Biowest, Nuaillé, France) and 1% penicillin-streptomycin (Life Technologies) at 37°C in 95% air and 5% CO_2_ [23]. Forming colonies were separated by incubation with Accutase (Funakoshi Co., Ltd., Tokyo, Japan), and isolated cells were transferred to passage cultures with DMEM supplemented by 10% FBS and 1% penicillin-streptomycin. The culture medium was changed every 2 days. Cells at passages 3–9 were used in subsequent experiments.

### 2.2. FACS Analysis

Cells were harvested with Accutase and fixed in 4% paraformaldehyde. Cells were centrifuged at 1,500 ×g for 5 min and resuspended at 5 × 10^6^ cells/mL in PBS containing 0.5% bovine serum albumin (BSA). Aliquots containing 10^5^ cells were incubated with individual phycoerythrin- (PE-) conjugated antibodies or isotype control PE-conjugated IgG*κ* for 30 min at room temperature and then washed in PBS supplemented with 3% FBS. Samples were analyzed using a FACS Aria flow cytometer (Becton Dickinson, Franklin Lakes, NJ, USA) and the data were analyzed using CELLQUEST software (Becton Dickinson). The following monoclonal antibodies were used: PE-conjugated antibodies against CD73 (mouse IgG1*κ*; Biolegend, San Diego, CA, USA), CD90 (mouse IgG1*κ*; Biolegend), CD105 (mouse IgG1*κ*; Biolegend), and CD166 (mouse IgG1*κ*; Santa Cruz Biotechnology, Texas, USA). PE-conjugated isotype control mouse IgG1*κ* (Biolegend) was used as the control.

### 2.3. *MSX1* Knockdown

MSX1 siRNA oligonucleotides (s8999 and s224066) were purchased from Life Technologies. The sequences are 5′-GCAUUUAGAUCUACACUCUtt-3′ (sense) and 5′-AGAGUGUAGAUCUAAAUGCta-3′ (antisense) for s8999 and 5′-GCAAGA AAAGCGCAGAGAAtt-3′ (sense) and 5′-UUCUCUGCGCUUUUCUUGCct-3′ (antisense) for s224066. Silencer select negative control #1 siRNA (Life Technologies) was used as the control.

Human DPSCs were seeded at 5 × 10^4^ cells/well in 24-multiwell plates coated with type I collagen with 0.5 mL DMEM supplemented with 10% FBS. After 24 h, siRNA was transfected into cells with Lipofectamine 2000 (Life Technologies) and cells were incubated for an additional 48 h.

### 2.4. Osteogenic Differentiation of hDPSCs and Alizarin Red Staining

After the cultures became confluent, hDPSCs were incubated with 0.5 mL of DMEM supplemented with 10% FBS, 10 mM *β*-glycerophosphate (Tokyo Chemical Industry Co., Ltd., Tokyo, Japan), 50 *μ*g/mL ascorbic acid 2-phosphate (Sigma), 2 mM L-glutamine (Sigma), 100 nM dexamethasone (Sigma), and 1% penicillin/streptomycin (osteogenesis-induction medium) as described [15]. For evaluation of calcification, cells incubated with osteogenesis-induction medium for 14 days were fixed at room temperature in 95% ethanol for 10 min and stained with 1% alizarin red S for 30 min. The cell-matrix layers were washed 6 times with sterile water.

### 2.5. Alkaline Phosphatase Activity

Human DPSCs were washed twice with saline and homogenized ultrasonically with 1% NP-40 in saline. Alkaline phosphatase activity was determined using Lab Assay ALP (Wako, Osaka, Japan). DNA concentration was determined with the Quant-iT*™* PicoGreen dsDNA Assay Kit (Life Technologies) to calculate alkaline phosphatase activity/*μ*g DNA.

### 2.6. Reverse Transcription-Quantitative Polymerase Chain Reaction (RT-qPCR)

Total RNA was isolated and cDNA was synthesized as described [15]. The cDNA samples were amplified using Universal PCR Master Mix (Life Technologies) with primers (Table 1) and TaqMan probes were purchased from Roche Diagnostics (Basel, Switzerland). GAPDH primers/probe set was used for normalization. After amplification of DNA, expression levels were determined with the ABI prism 7900 HT sequence detection system (Life Technologies).

### 2.7. DNA Microarray

After the induction of osteogenic differentiation for 4 days, total RNA was isolated from MSX1-knockdown and control hDPSCs using TRIzol (Life Technologies, Japan) and an RNeasy Mini Kit (Qiagen, Chatsworth, CA). DNA microarray analysis was performed using the SurePrint G3 Human GE 8 × 60 K v2 Microarray (Agilent Technologies, Santa Clara, CA, USA). Raw data were standardized by the global median normalization method using GeneSpring (Silicon Genetics, Redwood City, CA, USA). The raw data were deposited in the Gene Expression Omnibus database (GSE69992).

### 2.8. Statistical Analysis

Results are expressed as mean ± SD. Differences between two groups were analyzed by two-way ANOVA with Tukey's* post hoc* test for multiple comparisons. In all analyses, *P* < 0.05 indicated statistically significant differences between values.

## 3. Results 

### 3.1. Mesenchymal Stem Cell Markers Expressed in Cultured hDPSCs

**Figure 1:**
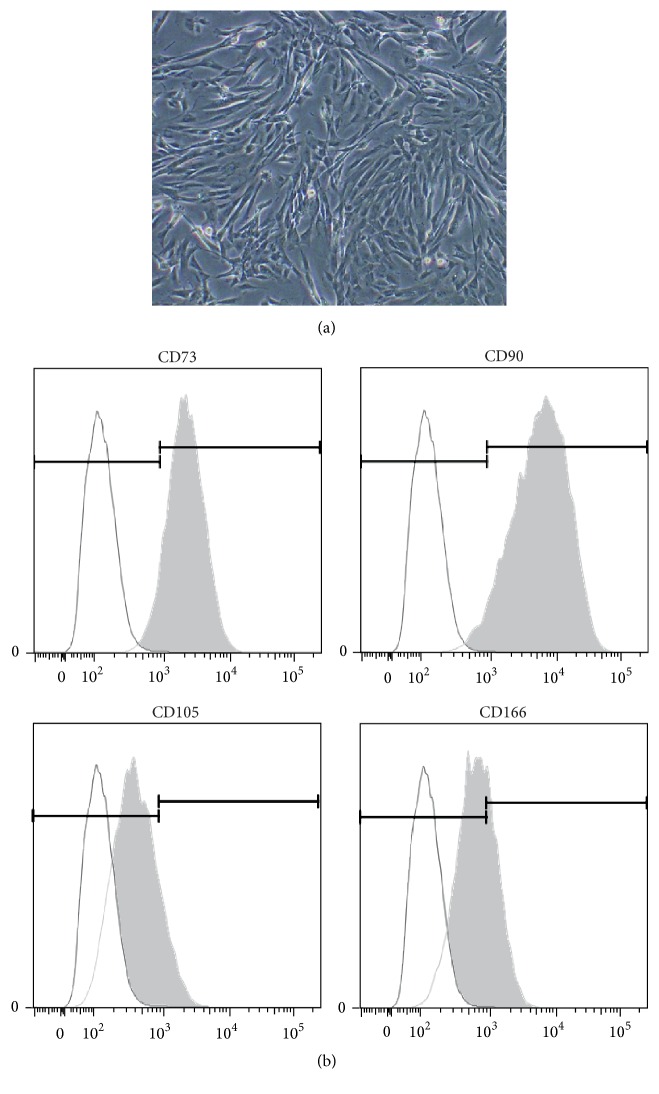
(a) A microscope image of cultured hDPSCs isolated from human primary teeth with no induction. (b) Positive expression of several cell surface antigens for mesenchymal stem cells, including CD73, CD90, CD105, and CD166, in hDPSCs. Similar cell surface antigen expression pattern was obtained with hDPSCs isolated from different donors (data not shown).

Human DPSCs from postnatal human primary teeth were used to explore the functional role of MSX1. These cells exhibited a fibroblastic shape (Figure 1(a)) and showed expression of mesenchymal stem cell surface markers CD73 (>90%), CD90 (>90%), CD105 (>10%), and CD166 (>30%) (Figure 1(b)) as expected from previous studies [24].

### 3.2. *MSX1* Knockdown Abolishes Osteogenic Differentiation of hDPSCs

**Figure 2:**
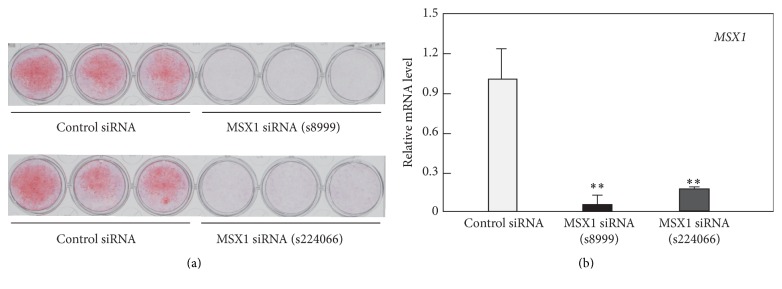
Effects of* MSX1* knockdown on calcification of hDPSCs. hDPSCs were transfected with either MSX1 siRNA (s8999 or s224066) or control siRNA and incubated for 2 days in growth medium before the cultures became confluent. Thereafter, the cultures were exposed to osteogenesis-induction medium. (a) The calcified matrix was stained with alizarin red on day 14. (b)* MSX1* mRNA level was quantified by RT-qPCR at 48 h after transfection. Values are averages ±SD for three cultures. ^*∗∗*^ *P* < 0.01.

Human DPSCs were transfected with two different siRNAs for* MSX1* or control siRNA and then exposed to osteogenesis-induction medium. Both siRNA oligonucleotides targeting* MSX1* (s8999 and s224066) abolished* MSX1* mRNA expression at 48 h and subsequent matrix calcification on day 14 (Figure 2). We selected* MSX1* siRNA (s8999) for subsequent studies.

**Figure 3:**
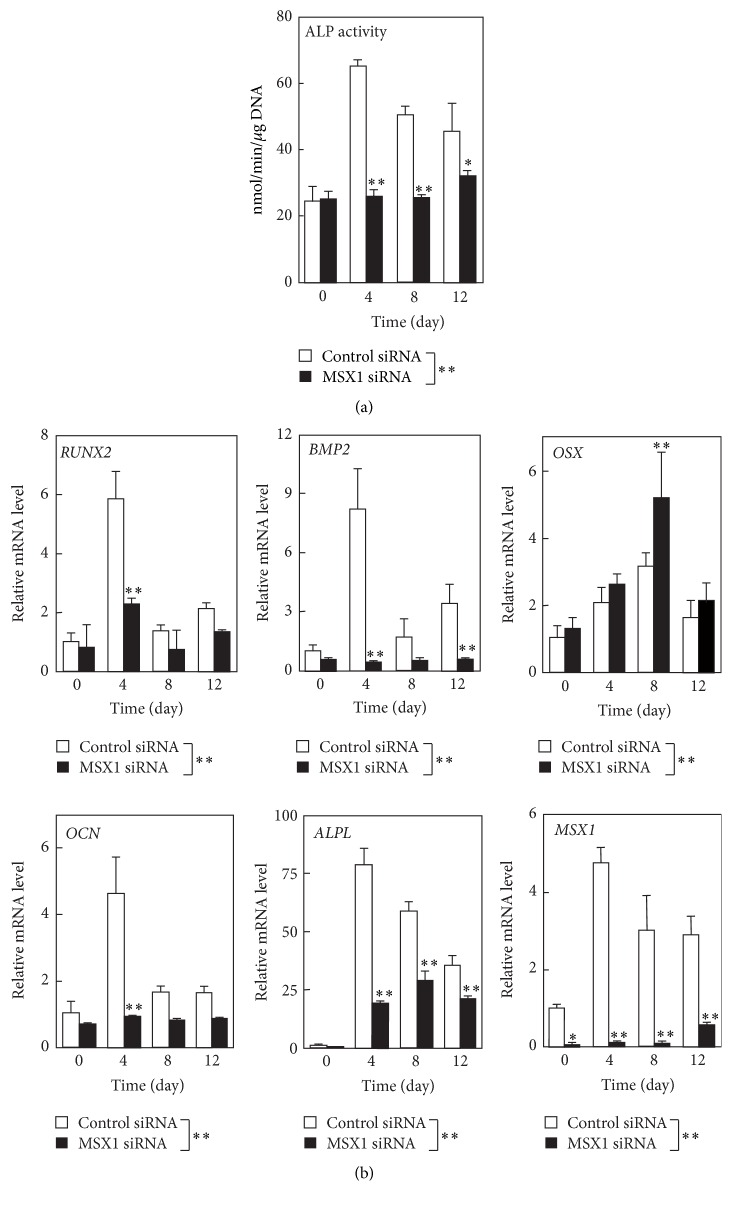
Effects of* MSX1* knockdown on the expression of osteogenic markers in hDPSCs. (a) Alkaline phosphatase activity in cultures was determined on days 0–12 after exposure to osteogenesis-induction medium. (b) The mRNA levels of osteoblast-related genes, including* RUNX2*,* BMP2*,* OSX*,* OCN*, and* ALPL*, along with the* MSX1* mRNA level were quantified by RT-qPCR on the indicated days. Values are averages ±SD for three cultures. ^*∗*^ *P* < 0.05; ^*∗∗*^ *P* < 0.01.

Next, we examined the effect of* MSX1* knockdown on alkaline phosphatase activity and the expression of osteoblast-related genes in hDPSCs after the onset of osteogenesis (Figure 3). In hDPSCs transfected with control siRNA, alkaline phosphatase activity and* RUNX2*,* BMP2*, osterix* (OSX)*, osteocalcin (*OCN*; also known as* BGLAP*), and alkaline phosphatase liver type* (ALPL)* mRNA levels increased on days 4–12 after the onset of differentiation.* MSX1* mRNA levels also increased on days 4–12. However,* MSX1* knockdown abolished the induction of alkaline phosphatase activity (Figure 3(a)) and the increases in* ALPL*,* RUNX2*,* BMP2*,* OCN*, and* MSX1* mRNA levels, although it further increased* OSX* mRNA levels (Figure 3(b)). It should be noted that the incubation with MSX1 siRNA abolished MSX1 expression at least until day 12 after the onset of osteogenic differentiation.

**Figure 4:**
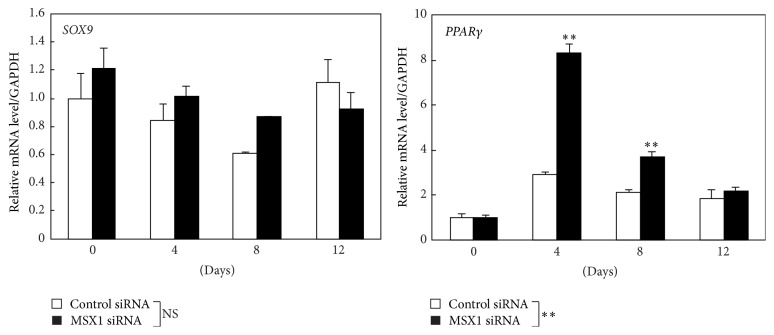
Effects of* MSX1* knockdown on the expression of the master genes of chondrogenesis and adipogenesis in hDPSCs. The mRNA levels of* SOX9* and* PPARγ* in hDPSCs transfected with MSX1 siRNA or control siRNA were quantified by RT-qPCR on the indicated days. Values are averages ±SD for 3 cultures. ^*∗∗*^ *P* < 0.01; NS: not significant.

Next, we examined whether MSX1 knockdown might influence the expression of other master genes including a master regulator of chondrogenesis* SOX9* and a master regulator of adipogenesis* PPARγ* (Figure 4). In control hDPSCs, no significant changes in the expressions of* SOX9* and* PPARγ* were observed after the exposure to osteogenesis-induction medium. Under these conditions, MSX1 knockdown increased the expression of* PPARγ* on days 4–8, although it had little effect on the expression level of* SOX9*.

### 3.3. *MSX1* Knockdown Downregulated and Upregulated a Variety of Genes

To characterize the effects of* MSX1* knockdown on osteogenic differentiation, we performed DNA microarray analyses on day 4 after exposure to osteogenesis-induction medium.* MSX1* knockdown decreased and increased mRNA levels of 2923 and 3480 genes, respectively, which were selected with cut-off values of >1.5-fold change and *t*-test *P* < 0.05. Tables 2 and 3 show lists of downregulated and upregulated genes in* MSX1*-knockdown hDPSCs, respectively.

To understand MSX1 actions in hDPSCs differentiating into osteoblasts, we performed a gene-set approach using the 2923 downregulated and 3480 upregulated genes. The WikiPathways analysis showed that the* MSX1* knockdown downregulated various genes involved in focal adhesion, endochondral ossification, integrin-mediated cell adhesion, matrix metalloproteinases, calcium regulation, and insulin signaling (Table 4), whereas it upregulated genes involved in sterol regulatory element-binding protein (SREBP) signaling, cholesterol biosynthesis, adipogenesis, and fatty acid biosynthesis (Table 5). These findings revealed that MSX1 regulates various cellular processes in hDPSCs differentiating into osteoblasts.

### 3.4. *MSX1* Knockdown Upregulates Cholesterol Synthesis-Related Genes

**Figure 5:**
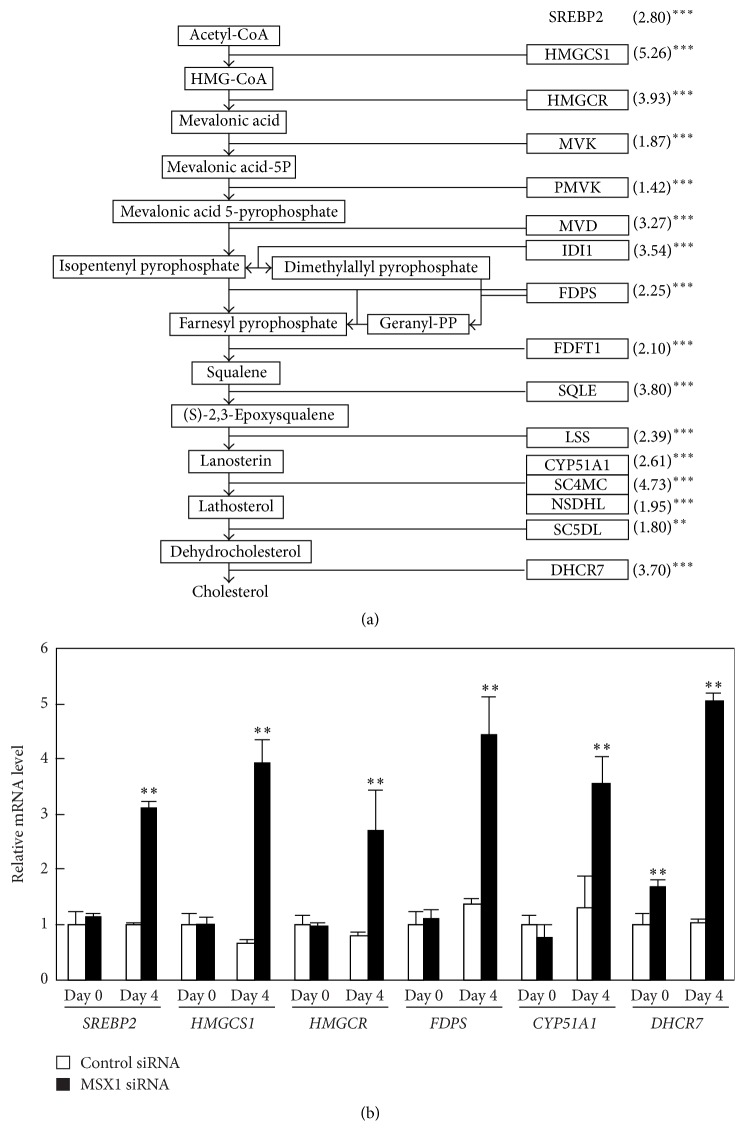
Effects of* MSX1* knockdown on the expression of cholesterol synthesis-related genes, which are direct targets for SREBP2, in hDPSCs. (a) The cholesterol biosynthesis pathway is shown and the bold frames indicate target genes for the master transcriptional factor SREBP2. Microarray analysis indicates all genes involved in cholesterol synthesis are upregulated by* MSX1* knockdown. The numbers in brackets represent the fold changes in gene expression in* MSX1*-knockdown cells as compared with the control cells. (b) The mRNA levels of genes relevant to cholesterol synthesis, including* SREBP2*,* HMGCS1*,* HMGCR*,* FDPS*,* CYP51A1*, and* DHCR7*, were quantified by RT-qPCR analysis. Values are averages ±SD for three cultures. ^*∗∗*^ *P* < 0.01; ^*∗∗∗*^ *P* < 0.001.

In* MSX1*-knockdown hDPSCs, “SREBP signaling” and “cholesterol biosynthesis” were the top 1st and 3rd upregulated gene sets, respectively (Table 5). The SREBP2 master transcriptional factor regulates the expression of all genes encoding enzymes in cholesterol synthesis pathway [25]. Because cholesterol synthesis is closely linked with osteoblast differentiation [17], we examined the effect of* MSX1* knockdown on the expression of these genes. DNA microarray analyses showed that all cholesterol synthesis-related genes, including* SREBP2*, were significantly upregulated by* MSX1* knockdown on day 4 (Figure 5(a)). Quantitative RT-PCR analyses confirmed that* MSX1* knockdown increased* SREBP2*, 3-hydroxy-3-methylglutaryl-CoA synthase 1* (HMGCS1)*, HMG-CoA reductase* (HMGCR)*, farnesyl diphosphate synthase* (FDPS)*, Cytochrome P450 Family 51 Subfamily A Polypeptide 1* (CYP51A1)*, and 7-dehydrocholesterol reductase* (DHCR7)* mRNA levels (Figure 5(b)).

## 4. Discussion

Previous studies showed that mouse MSX1 was implicated in craniofacial bone development [1, 2, 4]. In mouse embryos, MSX1 suppresses precocious differentiation and calcification in dental mesenchymal cells and maintains these cells in a proliferative state to ensure subsequent craniofacial and tooth development [6, 26]. High levels of osteoblast number, cell proliferation, and apoptosis in MSX1 transgenic mice suggest that MSX1 modulates mouse craniofacial bone modeling [4]. However, the role of MSX1 in human cells remains poorly understood. In the present study, we demonstrated that MSX1 plays an essential role in osteogenic differentiation of hDPSCs.

In human DPSC cultures,* MSX1* knockdown resulted in suppressed expression of* RUNX2*,* ALPL*,* BMP2*, and* OCN*. These results demonstrate that MSX1 modulated the major signaling/transcriptional pathways regulating hard tissue differentiation to enhance osteogenic potential of hDPSCs. However,* MSX1* knockdown unexpectedly increased the mRNA level of* OSX*, another transcriptional factor involved in osteoblast maturation. This indicates MSX1 does not activate the entire osteogenesis program, perhaps because MSX1 cooperates with other transcription factors to fully control osteogenesis.* MSX1* knockdown enhanced* PPARγ* expression under the osteogenesis-induced condition, suggesting that MSX1 negatively regulates adipogenic differentiation. MSX1 may direct hDPSCs into the osteoblast lineage by preventing them from differentiating into the adipogenic lineage.* MSX1* knockdown also resulted in downregulation of various genes involved in focal adhesion, integrin-mediated cell adhesion, matrix metalloproteinases, calcium regulation, insulin signaling, and other processes. The extensive effect of* MSX1* knockdown on the entire gene expression profile emphasizes a crucial role of MSX1 in hDPSCs undergoing differentiation into osteoblasts.

Bidirectional transcription of the* Msx1* gene has been previously reported [27–29]. In embryonic and newborn mice, sense and antisense* Msx1* transcripts are differently expressed during development. In 705IC5 mouse odontoblasts, overexpression of* Msx1* antisense RNA decreased the expression of* Msx1* sense transcript, whereas overexpression of* Msx1* sense RNA increased* Msx1* antisense transcript. Thus, expression of mouse* Msx1* is controlled by the balance of the two transcripts. In our experiments, however,* MSX1* antisense transcript was not detected during osteogenic differentiation of hDPSCs irrespective of siRNA knockdown of* MSX1* (data not shown). The presence of* MSX1* antisense transcript in humans has so far been reported only in the embryo. Therefore,* Msx1* antisense RNA does not seem to be involved in the MSX1 expression in hDPSCs. Under these conditions, the expression of* Msx1* sense transcripts was markedly depressed in hDPSCs after treatment with MSX1 siRNA, indicating that the knockdown experiments worked appropriately regardless of the presence or absence of the* Msx1* antisense transcript.

MSX2, a paralog of MSX1, has been shown to enhance osteogenic differentiation of various mesenchymal cells, including C3H10T1/2 cells and aorta myofibroblasts [30–32]. MSX1 and MSX2 activate aortic adventitial osteoprogenitors via overlapping yet distinct mechanisms [33]. MSX2, unlike MSX1, enhances* OSX* expression without an increase in* RUNX2* expression in aortic myofibroblasts [30], suggesting distinct actions of MSX1 and MSX2 in osteoblast differentiation.

The molecular mechanism by which MSX1 activates the differentiation program remains unclear. MSX1 regulates transcriptional activity of target genes either by directly binding to the specific DNA MSX1-binding motif (C/GTAATTG) or through interactions with other transcriptional regulators. Interestingly, MSX1 binds to various transcriptional regulators, including Sp1, Sp3, Dlx3, Dlx5, PAX3, PAX9, BarH-like homeobox 1/BARX1, and PIAS1 [34–37]. Depending on the partner in the complex, MSX1 activates or represses transcription in the MSX1-interacting network of transcription factors [26, 38]. Moreover, MSX1 modifies chromatin structure near target genes by histone methylation [35, 39]. The interactions of MSX1 with various transcriptional regulators may account for the extensive changes in the expression levels of many genes (~6400) by* MSX1* knockdown.

Statins, drugs for hyperlipidemia, enhance osteogenic differentiation of various mesenchymal cells, including osteoblast precursor cells, mesenchymal stem cells, and DPSCs, by inhibiting the synthesis of farnesyl pyrophosphate, decreasing cellular cholesterol, and activating the Ras-PI3K-Akt/MAPK signaling pathway, thereby increasing the expression of BMP2 and RUNX2 [17], although the underlying mechanisms are still controversial. Statins also suppress osteoclast function and enhance mandibular bone formation* in vivo* [40]. Interestingly, a previous study showed that simvastatin induces odontoblast differentiation of hDPSCs* in vitro* and* in vivo* [22]. However, no studies have shown the involvement of transcription factor(s) in the control of cholesterol synthesis during osteoblast differentiation. Here we found for the first time that MSX1 suppresses the entire cholesterol synthesis pathway in osteoblast differentiating hDPSCs by repressing* SREBP2* and other related genes. This suppression of cholesterol synthesis may facilitate osteoblast differentiation. It is also interesting to note that various mutations in the cholesterol synthesis pathway, including 7-dehydrocholesterol reductase* (DHCR7)*, cause craniofacial anomalies including cleft palate, suggesting the role of cholesterol synthesis in craniofacial development [41].

In conclusion, here we revealed for the first time that MSX1 is indispensable for osteoblast-like differentiation and calcification in hDPSCs derived from deciduous teeth. Furthermore, MSX1 was found to modulate a wide variety of genes, including cholesterol synthesis-related genes, during osteogenic differentiation of hDPSCs. We have not examined the effects of MSX1 siRNA in definitive teeth, although MSX1 may also function as a positive regulator of osteogenesis in definitive teeth as MSX1 mRNA levels are high in both definitive and deciduous teeth [15]. Our findings will provide new insights into the role of MSX1 in development and repair of teeth and may be useful in DPSC-based regenerative therapy.

## Figures and Tables

**Table 1:** Primer and probe sequences used for RT-qPCR.

Gene	Primer (5′ → 3′)	Probe
MSX1 (sense)	F: CTCGTCAAAGCCGAGAGC	Roche Universal Probe # 7
	R: CGGTTCGTCTTGTGTTTGC	
MSX1 (antisense)	F: GCCAGCCCTCTTAGAAACAG	Roche Universal Probe # 50
	R: AATAAAGCAGCCCCTCGTTC	
RUNX2	F: CAGTGACACCATGTCAGCAA	Roche Universal Probe # 66
	R: GCTCACGTCGCTCATTTTG	
BMP2	F: CGGACTGCGGTCTCCTAA	Roche Universal Probe # 49
	R: GGAAGCAGCAACGCTAGAAG	
OSX	F: CAGCAGCTAAACTTGGAAGGA	Roche Universal Probe # 76
	R: TGCTTTCGCTTGTCTGAGTC	
OCN	F: GCCTCCTGAAAGCCGATGT	5′-CCAACTCGTCACAGTCCGGATTGAGCT-3′
	R: AAGAGACCCAGGCGCTACCT	
ALPL	F: TCACTCTCCGAGATGGTGGT	Roche Universal Probe # 12
	R: GTGCCCGTGGTCAATTCT	
SOX9	F: GTACCCGCACTTGCACAAC	Roche Universal Probe # 61
	R: TCTCGCTCTCGTTCAGAAGTC	
PPAR*γ*	F: GACAGGAAAGACAACAGACAAATC	Roche Universal Probe # 7
	R: GGGGTGATGTGTTTGAACTTG	
SREBP2	F: GCCCTGGAAGTGACAGAGAG	Roche Universal Probe # 21
	R: TGCTTTCCCAGGGAGTGA	
HMGCS1	F: TCTGTCTACTGCAAAAAGATCCAT	Roche Universal Probe # 59
	R: TGAAGCCAAAATCATTCAAGG	
HMGCR	F: GTTCGGTGGCCTCTAGTGAG	Roche Universal Probe # 65
	R: GCATTCGAAAAAGTCTTGACAAC	
FDPS	F: GGCCACTCCAGAACAGTACC	Roche Universal Probe # 75
	R: CCTCATATAGCGCCTTCACC	
CYP51A1	F: TGCAGATTTGGATGGAGGTT	Roche Universal Probe # 64
	R: CCTTGATTTCCCGATGAGC	
DHCR7	F: GCCATGGTCAAGGGCTAC	Roche Universal Probe # 60
	R: TTGTAAAAGAAATTGCCTGTGAAT	

MSX1: msh homeobox 1; RUNX2: runt-related transcription factor-2; BMP2: bone morphogenetic protein-2; OSX: osterix; OCN: osteocalcin; ALPL: alkaline phosphatase liver type; SOX9: SRY- (sex determining region Y-) box 9; PPAR*γ*: peroxisome proliferator activated receptor gamma; SREBP2: sterol regulatory element-binding protein 2; HMGCS1: 3-hydroxy-3-methylglutaryl-CoA synthase 1; HMGCR: HMG-CoA reductase; FDPS: farnesyl diphosphate synthase; CYP51A1: Cytochrome P450 Family 51 Subfamily A Polypeptide 1; DHCR7: 7-dehydrocholesterol reductase; F: forward; R: reverse.

**Table 2:** The list of downregulated genes in MSX1-knockdown cells (top 50).

Gene symbol	Gene name	Gene ID	Fold change
C11orf96	Chromosome 11 open reading frame 96	NM_001145033	92.53
JAM2	Junctional adhesion molecule 2	NM_021219	70.90
MLC1	Megalencephalic leukoencephalopathy with subcortical cysts 1	NM_015166	65.56
NPPC	Natriuretic peptide C	NM_024409	54.40
CPXM1	Carboxypeptidase X (M14 family), member 1	NM_019609	51.08
EFCC1	EF-hand and coiled-coil domain containing 1	NM_024768	44.56
SFRP4	Secreted frizzled-related protein 4	NM_003014	43.96
JAM2	Junctional adhesion molecule 2	NM_021219	38.31
LOC200772	Uncharacterized LOC200772	NR_033841	34.41
S100A8	S100 calcium binding protein A8	NM_002964	31.72
JAM2	Junctional adhesion molecule 2	NM_001270408	31.42
CPXM1	Carboxypeptidase X (M14 family), member 1	NM_019609	29.44
SFRP4	Secreted frizzled-related protein 4	NM_003014	28.11
KIT	v-kit Hardy-Zuckerman 4 feline sarcoma viral oncogene homolog	NM_000222	27.48
CLCA2	Chloride channel accessory 2	NM_006536	24.88
LINC00473	Long intergenic nonprotein coding RNA 473	NR_026860	24.34
ST8SIA4	ST8 alpha-N-acetyl-neuraminide alpha-2,8-sialyltransferase 4	NM_005668	24.16
TEX29	Testis expressed 29	NM_152324	23.52
PTGDR2	Prostaglandin D2 receptor 2	NM_004778	23.31
CXCL14	Chemokine (C-X-C motif) ligand 14	NM_004887	22.63
HS6ST2	Heparan sulfate 6-O-sulfotransferase 2	NM_001077188	22.61
CHST15	Carbohydrate (N-acetylgalactosamine 4-sulfate 6-O) Sulfotransferase 15	NM_015892	21.84
PIANP	PILR alpha associated neural protein	NM_153685	21.67
SNAP25	Synaptosomal-associated protein, 25 kDa	NM_003081	21.55
CBLN2	Cerebellin 2 precursor	NM_182511	21.30
FRAS1	Fraser syndrome 1	NM_025074	21.24
SECTM1	Secreted and transmembrane 1	NM_003004	20.76
NFE2	Nuclear factor, erythroid 2	NM_006163	20.46
GABBR2	Gamma-aminobutyric acid (GABA) B receptor, 2	NM_005458	19.22
MSX1	Msh homeobox 1	NM_002448	19.08
SCARA5	Scavenger receptor class A, member 5 (putative)	NM_173833	18.79
PRSS35	Protease, serine, 35	NM_153362	18.60
WNT2B	Wingless-type MMTV integration site family, member 2B	NM_004185	17.95
BMP2	Bone morphogenetic protein-2	NM_001200	17.63
NDRG4	NDRG family member 4	NM_022910	17.39
CRTAM	Cytotoxic and regulatory T cell molecule	NM_019604	16.80
RASL12	RAS-like, family 12	NM_016563	15.91
THBD	Thrombomodulin	NM_000361	15.26
CHST1	Carbohydrate (keratan sulfate Gal-6) sulfotransferase 1	NM_003654	14.90
DIO3OS	DIO3 opposite strand/antisense RNA (head to head)	NR_002770	14.81
CCR1	Chemokine (C-C motif) receptor 1	NM_001295	14.81
TMEM35	Transmembrane protein 35	NM_021637	14.79
HAS1	Hyaluronan synthase 1	NM_001523	14.68
SCN1B	Sodium channel, voltage-gated, type I, beta subunit	NM_199037	14.34
ADAMTS17	ADAM metallopeptidase with thrombospondin type 1 motif, 17	NM_139057	14.12
GALNT15	UDP-N-acetyl-alpha-D-galactosamine:polypeptide N-acetylgalactosaminyltransferase 15	NM_054110	13.86
RHOH	Ras homolog family member H	NM_004310	13.80
GPR68	G protein-coupled receptor 68	NM_003485	13.63
TAC3	Tachykinin 3	NM_013251	13.37
MIR1247	MicroRNA 1247	AF469204	13.13

**Table 3:** The list of upregulated genes in MSX1-knockdown cells (top 50).

Gene symbol	Gene name	Gene ID	Fold change
MLXIPL	MLX interacting protein-like	NM_032951	74.63
CRLF1	Cytokine receptor-like factor 1	NM_004750	73.44
ATP1A2	ATPase, Na+/K+ transporting, alpha 2 polypeptide	NM_000702	69.84
MAP2	Microtubule-associated protein 2	NM_002374	62.64
PRODH	Proline dehydrogenase (oxidase) 1	NM_016335	50.32
LONRF3	LON peptidase N-terminal domain and ring finger 3	NM_001031855	30.84
ERICH2	Glutamate-rich 2	XM_001714892	29.67
DMD	Dystrophin	NM_004010	27.91
SAA1	Serum amyloid A1	NM_000331	26.65
CLDN20	Claudin 20	NM_001001346	24.19
DMD	Dystrophin	NM_004021	21.88
PLIN4	Perilipin 4	NM_001080400	21.64
RLBP1	Retinaldehyde binding protein 1	NM_000326	21.22
SAA2	Serum amyloid A2	NM_030754	20.98
PARD6B	Par-6 family cell polarity regulator beta	NM_032521	19.56
ANO3	Anoctamin 3	NM_031418	17.58
KCNJ16	Potassium inwardly rectifying channel, subfamily J, member 16	NM_170741	17.41
LOC284561	Uncharacterized LOC284561	XR_110828	16.52
USP53	Ubiquitin specific peptidase 53	NM_019050	16.47
CLGN	Calmegin	NM_004362	16.35
USP53	Ubiquitin specific peptidase 53	NM_019050	16.15
PLCE1-AS1	PLCE1 antisense RNA 1	NR_033969	15.15
KLHDC7B	Kelch domain containing 7B	NM_138433	14.44
PSG9	Pregnancy specific beta-1-glycoprotein 9	NM_002784	14.23
ERICH2	Glutamate-rich 2	XM_001714892	13.66
ANKRD1	Ankyrin repeat domain 1 (cardiac muscle)	NM_014391	13.58
PDE6A	Phosphodiesterase 6A, cGMP-specific, rod, alpha	NM_000440	13.23
COL4A4	Collagen, type IV, alpha 4	NM_000092	12.98
PLAC8	Placenta-specific 8	NM_016619	12.85
BEST2	Bestrophin 2	NM_017682	12.72
IP6K3	Inositol hexakisphosphate kinase 3	NM_054111	12.22
DNAH2	Dynein, axonemal, heavy chain 2	NM_020877	12.07
INHBB	Inhibin, beta B	NM_002193	11.63
LOC100506544	Uncharacterized LOC100506544	AK057177	10.9
TMEM125	Transmembrane protein 125	NM_144626	10.57
ORM2	Orosomucoid 2	NM_000608	10.51
PCSK9	Proprotein convertase subtilisin/kexin type 9	NM_174936	10.17
CD200	CD200 molecule	NM_001004196	9.99
MFSD2A	Major facilitator superfamily domain containing 2A	NM_001136493	9.85
IL8	Interleukin 8	NM_000584	9.80
FMO6P	Flavin containing monooxygenase 6 pseudogene	NR_002601	9.60
C6	Complement component 6	NM_000065	9.54
DSCR8	Down syndrome critical region gene 8	NR_026838	9.23
LOC648149	Uncharacterized LOC648149	AK123349	9.19
GPR18	G protein-coupled receptor 18	NM_005292	9.14
ORM1	Orosomucoid 1	NM_000607	9.04
GATA3	GATA binding protein 3	NM_001002295	9.03
KCNB1	Potassium voltage-gated channel, Shab-related subfamily, member 1	NM_004975	8.97
OCA2	Oculocutaneous albinism II	NM_000275	8.90
PDZRN4	PDZ domain containing ring finger 4	NM_013377	8.86

**Table 4:** The WikiPathways analysis selected gene sets significantly downregulated in MSX1-knockdown cells.

Ranking	Pathway	*P* value	Gene counts (gene number of pathway)
1	Focal adhesion	4.20*E* − 09	36 (188)
2	IL-4 signaling pathway	2.55*E* − 05	13 (55)
3	Endochondral ossification	6.27*E* − 05	14 (64)
4	Muscle cell tarbase	7.95*E* − 05	41 (336)
5	Integrin-mediated cell adhesion	8.22*E* − 05	18 (99)
6	Matrix metalloproteinases	9.72*E* − 05	9 (31)
7	Regulation of toll-like receptor signaling pathway	1.12*E* − 04	22 (150)
8	Calcium regulation in the cardiac cell	1.30*E* − 04	23 (149)
9	MicroRNAs in cardiomyocyte hypertrophy	3.35*E* − 04	15 (105)
10	Insulin signaling	4.14*E* − 04	23 (161)

**Table 5:** The WikiPathways analysis selected gene sets significantly upregulated in MSX1 knockdown cells.

Ranking	Pathway	*P* value	Gene counts (gene number of pathway)
1	SREBP signalling	<1.00*E* − 44	24 (50)
2	Lymphocyte tarbase	<1.00*E* − 44	78 (420)
3	Cholesterol biosynthesis	1.86*E* − 17	16 (17)
4	Muscle cell tarbase	1.78*E* − 10	65 (336)
5	Epithelium tarbase	9.47*E* − 10	52 (278)
6	Folate metabolism	1.47*E* − 09	22 (68)
7	Adipogenesis	3.19*E* − 08	30 (131)
8	Leukocyte tarbase	1.47*E* − 07	28 (128)
9	Fatty acid biosynthesis	1.73*E* − 06	10 (22)
10	SREBF and miR33 in cholesterol and lipid homeostasis	8.09*E* − 06	8 (18)

## References

[1] Satokata I., Maas R. (1994). Msx1 deficient mice exhibit cleft palate and abnormalities of craniofacial and tooth development. *Nature Genetics*.

[2] Maas R., Chen Y. P., Bei M., Woo I., Satokata I. (1996). The role of Msx genes in mammalian development. *Annals of the New York Academy of Sciences*.

[3] Orestes-Cardoso S. M., Nefussi J. R., Hotton D. (2001). Postnatal Msx1 expression pattern in craniofacial, axial, and appendicular skeleton of transgenic mice from the first week until the second year. *Developmental Dynamics*.

[4] Nassif A., Senussi I., Meary F. (2014). Msx1 role in craniofacial bone morphogenesis. *Bone*.

[5] Mackenzie A., Leeming G. L., Jowett A. K., Ferguson M. W. J., Sharpe P. T. (1991). The homeobox gene Hox 7.1 has specific regional and temporal expression patterns during early murine craniofacial embryogenesis, especially tooth development in vivo and in vitro. *Development*.

[6] Han J., Ito Y., Yeo J. Y., Sucov H. M., Maas R., Chai Y. (2003). Cranial neural crest-derived mesenchymal proliferation is regulated by Msx1-mediated p19INK4d expression during odontogenesis. *Developmental Biology*.

[7] Chen Y., Bei M., Woo I., Satokata I., Maas R. (1996). Msx1 controls inductive signaling in mammalian tooth morphogenesis. *Development*.

[8] Vieux-Rochas M., Bouhali K., Mantero S. (2013). BMP-mediated functional cooperation between *Dlx5;Dlx6* and *Msx1;Msx2* during mammalian limb development. *PLoS ONE*.

[9] Vastardis H., Karimbux N., Guthua S. W., Seidman J. G., Seidman C. E. (1996). A human MSX1 homeodomain missense mutation causes selective tooth agenesis. *Nature Genetics*.

[10] Yamaguchi S., Machida J., Kamamoto M. (2014). Characterization of novel MSX1 mutations identified in Japanese patients with nonsyndromic tooth agenesis. *PLoS ONE*.

[11] Nakatsuka R., Nozaki T., Uemura Y. (2010). 5-Aza-2'-deoxycytidine treatment induces skeletal myogenic differentiation of mouse dental pulp stem cells. *Archives of Oral Biology*.

[12] Ranganathan K., Lakshminarayanan V. (2012). Stem cells of the dental pulp. *Indian Journal of Dental Research*.

[13] Iohara K., Imabayashi K., Ishizaka R. (2011). Complete pulp regeneration after pulpectomy by transplantation of CD105+ stem cells with stromal cell-derived factor-1. *Tissue Engineering Part A*.

[14] Sakai K., Yamamoto A., Matsubara K. (2012). Human dental pulp-derived stem cells promote locomotor recovery after complete transection of the rat spinal cord by multiple neuro-regenerative mechanisms. *Journal of Clinical Investigation*.

[15] Fujii S., Fujimoto K., Goto N. (2015). Characteristic expression of MSX1, MSX2, TBX2 and ENTPD1 in dental pulp cells. *Biomedical Reports*.

[16] Li X., Cui Q., Kao C., Wang G.-J., Balian G. (2003). Lovastatin inhibits adipogenic and stimulates osteogenic differentiation by suppressing PPAR*γ*2 and increasing Cbfa1/Runx2 expression in bone marrow mesenchymal cell cultures. *Bone*.

[17] Ruan F., Zheng Q., Wang J. (2012). Mechanisms of bone anabolism regulated by statins. *Bioscience Reports*.

[18] Liu C., Wu Z., Sun H.-C. (2009). The effect of simvastatin on mRNA expression of transforming growth factor-beta1, bone morphogenetic protein-2 and vascular endothelial growth factor in tooth extraction socket. *International Journal of Oral Science*.

[19] Ayukawa Y., Yasukawa E., Moriyama Y. (2009). Local application of statin promotes bone repair through the suppression of osteoclasts and the enhancement of osteoblasts at bone-healing sites in rats. *Oral Surgery, Oral Medicine, Oral Pathology, Oral Radiology and Endodontology*.

[20] Pradeep A. R., Thorat M. S. (2010). Clinical effect of subgingivally delivered simvastatin in the treatment of patients with chronic periodontitis: a randomized clinical trial. *Journal of Periodontology*.

[21] Lee C.-T., Lee Y.-T., Ng H.-Y. (2012). Lack of modulatory effect of simvastatin on indoxyl sulfate-induced activation of cultured endothelial cells. *Life Sciences*.

[22] Okamoto Y., Sonoyama W., Ono M. (2009). Simvastatin induces the odontogenic differentiation of human dental pulp stem cells in vitro and in vivo. *Journal of Endodontics*.

[23] Gronthos S., Mankani M., Brahim J., Robey P. G., Shi S. (2000). Postnatal human dental pulp stem cells (DPSCs) in vitro and in vivo. *Proceedings of the National Academy of Sciences of the United States of America*.

[24] Karamzadeh R., Eslaminejad M. B., Aflatoonian R. (2012). Isolation, characterization and comparative differentiation of human dental pulp stem cells derived from permanent teeth by using two different methods. *Journal of Visualized Experiments*.

[25] Horton J. D., Goldstein J. L., Brown M. S. (2002). SREBPs: activators of the complete program of cholesterol and fatty acid synthesis in the liver. *The Journal of Clinical Investigation*.

[26] Feng X.-Y., Zhao Y.-M., Wang W.-J., Ge L.-H. (2013). Msx1 regulates proliferation and differentiation of mouse dental mesenchymal cells in culture. *European Journal of Oral Sciences*.

[27] Blin-Wakkach C., Lezot F., Ghoul-Mazgar S. (2001). Endogenous *Msx1* antisense transcript: *in vivo* and *in vitro* evidences, structure, and potential involvement in skeleton development in mammals. *Proceedings of the National Academy of Sciences of the United States of America*.

[28] Coudert A. E., Pibouin L., Vi-Fane B. (2005). Expression and regulation of the Msx1 natural antisense transcript during development. *Nucleic Acids Research*.

[29] Petit S., Meary F., Pibouin L. (2009). Autoregulatory loop of Msx1 expression involving its antisense transcripts. *Journal of Cellular Physiology*.

[30] Cheng S.-L., Shao J.-S., Charlton-Kachigian N., Loewy A. P., Towler D. A. (2003). MSX2 promotes osteogenesis and suppresses adipogenic differentiation of multipotent mesenchymal progenitors. *The Journal of Biological Chemistry*.

[31] Ichida F., Nishimura R., Hata K. (2004). Reciprocal roles of Msx2 in regulation of osteoblast and adipocyte differentiation. *The Journal of Biological Chemistry*.

[32] Ishii M., Merrill A. E., Chan Y.-S. (2003). *Msx2* and *Twist* cooperatively control the development of the neural crest-derived skeletogenic mesenchyme of the murine skull vault. *Development*.

[33] Cheng S.-L., Behrmann A., Shao J.-S. (2014). Targeted reduction of vascular *Msx1* and *Msx2* mitigates arteriosclerotic calcification and aortic stiffness in LDLR-deficient mice fed diabetogenic diets. *Diabetes*.

[34] Catron K. M., Zhang H., Marshall S. C., Inostroza J. A., Wilson J. M., Abate C. (1995). Transcriptional repression by Msx-1 does not require homeodomain DNA-binding sites. *Molecular and Cellular Biology*.

[35] Lee H., Habas R., Abate-Shen C. (2004). Msx1 cooperates with histone H1b for inhibition of transcription and myogenesis. *Science*.

[36] Wang J., Abate-Shen C. (2012). The Msx1 homeoprotein recruits G9a methyltransferase to repressed target genes in myoblast cells. *PLoS ONE*.

[37] Zhang H., Hu G., Wang H. (1997). Heterodimerization of Msx and Dlx homeoproteins results in functional antagonism. *Molecular and Cellular Biology*.

[38] Zhao M., Gupta V., Raj L., Roussel M., Bei M. (2013). A network of transcription factors operates during early tooth morphogenesis. *Molecular and Cellular Biology*.

[39] Wang J., Kumar R. M., Biggs V. J. (2011). The Msx1 homeoprotein recruits polycomb to the nuclear periphery during development. *Developmental Cell*.

[40] Lee Y., Schmid M. J., Marx D. B. (2008). The effect of local simvastatin delivery strategies on mandibular bone formation in vivo. *Biomaterials*.

[41] Wassif C. A., Zhu P., Kratz L. (2001). Biochemical, phenotypic and neurophysiological characterization of a genetic mouse model of RSH/Smith—Lemli—Opitz syndrome. *Human Molecular Genetics*.

